# Characterization of microbial communities from gut microbiota of hypercholesterolemic and control subjects

**DOI:** 10.3389/fcimb.2022.943609

**Published:** 2022-11-29

**Authors:** Cristian Morales, Gabriel Rojas, Camilo Rebolledo, Marcelo Rojas-Herrera, Raúl Arias-Carrasco, Sara Cuadros-Orellana, Vinicius Maracaja-Coutinho, Kathleen Saavedra, Pamela Leal, Fernando Lanas, Luis A. Salazar, Nicolás Saavedra

**Affiliations:** ^1^ Centro de Biología Molecular y Farmacogenética, Núcleo Científico-Tecnológico en Biorecursos BIOREN, Universidad de La Frontera, Temuco, Chile; ^2^ Tecnología Médica, Facultad de Salud, Universidad Santo Tomás, Temuco, Chile; ^3^ Centro de Genética y Genómica, Facultad de Medicina, Universidad del Desarrollo, Santiago, Chile; ^4^ Subdepartamento de Genética Molecular, Instituto de Salud Pública de Chile, Santiago, Chile; ^5^ Programa Institucional de Fomento a la Investigación, Desarrollo e Innovación, Universidad Tecnológica Metropolitana, Santiago, Chile; ^6^ Centro de Biotecnología de los Recursos Naturales, Facultad de Ciencias Agrarias y Forestales, Universidad Católica del Maule, Talca, Chile; ^7^ Advanced Center for Chronic Diseases - ACCDiS, Facultad de Química y Ciencias Farmacéuticas, Universidad de Chile, Santiago, Chile; ^8^ Departamento de Ciencias Agronómicas y Recursos Naturales, Facultad de Ciencias Agropecuarias y Medioambiente, Temuco, Chile; ^9^ Departamento de Medicina Interna, Facultad de Medicina, Universidad de La Frontera, Temuco, Chile

**Keywords:** gut microbiota, LDL cholesterol, LEfSe analysis, microbial signature, 16S rRNA sequencing

## Abstract

**Introduction:**

In recent years, several studies have evidenced the importance of the microbiome to host physiology as metabolism regulator, along with its potential role in triggering various diseases. In this study, we analyzed the gut microbiota in hypercholesterolemic (cases) and normocholesterolemic (controls) individuals to identify characteristic microbial signature for each condition.

**Methods:**

Stool samples were obtained from 57 adult volunteers (27 hypercholesterolemic and 30 controls). The taxonomic profiling of microbial communities was performed using high-throughput sequencing of 16S rRNA V3-V4 amplicons, followed by data analysis using Quantitative Insights Into Microbial Ecology 2 (QIIME2) and linear discriminant analysis (LDA) effect size (LEfSe).

**Results:**

Significant differences were observed in weight, height, body mass index (BMI) and serum levels of triglycerides, total cholesterol and low-density lipoprotein cholesterol (LDL-C) between the groups (p<0.05). LEfSe showed differentially abundant prokaryotic taxa (α=0.05, LDA score > 2.0) in the group of hypercholesterolemic individuals (*Methanosphaera*, *Rothia*, Chromatiales, Clostridiales, Bacillaceae and Coriobacteriaceae) and controls (*Faecalibacterium*, *Victivallis* and *Selenomonas)* at various taxonomic levels. In addition, through the application of Phylogenetic Investigation of Communities by Reconstruction of Unobserved States 2 (PICRUSt2), the predominance of pathways related to biosynthesis in hypercholesterolemic patients was established, compared to controls in which degradation pathways were predominant. Finally, in the analysis of co-occurrence networks, it was possible to identify associations between the microorganisms present in both studied groups.

**Conclusion:**

Our results point out to unique microbial signatures, which likely play a role on the cholesterol metabolism in the studied population.

## Introduction

The human microbiota is composed of cells from all three domains of life, being the prokaryotic assemblages the most well-known. It is estimated that humans harbor approximately 10 bacterial cells per eukaryotic cell, most of which are located in the gastrointestinal tract, where produce vitamins and process dietary components that are not digestible by another way (Bäckhed et al., 2005) . However, in recent years new calculations have been estimated the ratio between bacterial and human cells in 1:1, although the relevance of the microbiota and its impact on the body would not be altered (Sender et al., 2016). The imbalance of bacterial communities, known as dysbiosis, has been associated with the development of various diseases in recent years. Some examples are the association of the microbiome composition with cardiovascular diseases (CVD), diabetes mellitus, obesity, metabolic syndrome, and hypertension (Wu et al., 2015; Mazidi et al., 2016). Furthermore, an important association has been established between diet, gut microbiota composition, and CVDs (Mendelsohn and Larrick, 2013). Moreover, it has been suggested that the characterization of the gut microbiota could be a useful tool in the description of cardiovascular health status in the context of precision medicine (Antman and Loscalzo, 2016). In addition, other studies have associated the presence of gut microorganisms with trimethylamine, which originates from dietary carnitine and lecithin, contributing to development of CVDs (Mendelsohn and Larrick, 2013). Similarly, a possible relation of the intestinal microbiota with the metabolism of circulating lipids in blood should be considered, which could contribute to the development of CVDs, and which may be of interest for the treatment of these diseases (Mazidi et al., 2016) Currently, some studies show the association between microbial groups of the intestinal microbiota with classical lipid parameters and apolipoproteins A1 and B (Yun et al., 2020).

On the other hand, hypercholesterolemia is considered an important modifiable risk factor of CVD, since high levels of low-density lipoprotein cholesterol (LDL-C) are known to increase the risk of coronary artery disease (CAD). In addition, the pharmacological reduction of LDL-C also reduces the incidence and mortality by coronary events (Baigent et al., 2005; Yusuf et al., 2006). Studies in animal models have shown a relationship between the gut microbiota and disturbances in lipid metabolism. Mice lacking their gut microbiota present differences in lipid absorption resulting in variations of serum lipid levels (Wostmann, 1973). Other models have also confirmed the importance of bacteria in cholesterol metabolism, highlighting the relevance of microorganisms in this biological process and their therapeutic potential (Bourgin et al., 2020). Furthermore, specific bacterial groups detected in stool samples have been shown to have beneficial effects on lipid metabolism induced by dietary changes (Martinez et al., 2009). A previous study by our group, using the polymerase chain reaction followed by denaturing gradient gel electrophoresis (PCR-DGGE), showed that subjects with hypercholesterolemia and controls exhibit different gut microbiota profiles (Rebolledo et al., 2017).

Thus, the objective of this study was to describe the microbial communities differentially present in the gut microbiota of hypercholesterolemic and control individuals.

## Materials and methods

### Subjects

A case-control study was designed, including 27 hypercholesterolemic (cases) and 30 normocholesterolemic individuals (controls). LDL-C levels were considered to define these groups by using the criteria of the National Cholesterol Education Program (NCEP) (Grundy et al., 2004). Participants were recruited by the Center for Cardiological and Internal Medicine Studies of the Universidad de La Frontera (Temuco, Chile). Thus, individuals with concentrations of LDL-C greater than 4.16 mmol/L and less than 2.60 mmol/L were classified as hypercholesterolemic or control, respectively. The presence of inflammatory bowel disease, diabetes mellitus, class 2 and morbid obesity, use of lipid-lowering medications and treatment with antibiotics in the last six months were established as exclusion criteria. For this study, the participants were volunteers and signed an informed consent that was approved by the Scientific Ethics Committee of the Universidad de La Frontera, Temuco, Chile (protocol number 113_2016).

### Samples

Blood samples were collected by using standard venipuncture technique and serum was obtained to perform biochemical analysis. Additionally, stool samples were obtained from participants using a DNA/RNA Shield-Fecal Collection Tube (Zymo Research, USA). Then, samples were transported to the laboratory and stored at -80°C until DNA isolation.

### Anthropometric, clinical and biochemical parameters

Anthropometric and clinical parameters such as age, height, weight, body mass index (BMI), systolic blood pressure (SBP) and diastolic blood pressure (DBP) were recorded. Serum glucose, triglycerides, total cholesterol, high-density lipoprotein cholesterol (HDL-C) and LDL-C were quantified using enzymatic colorimetric methods. Quality measurements were controlled by using normal and pathological commercial serums (Wiener Lab., Argentina).

### DNA extraction and 16S rRNA gene sequencing

DNA was isolated from stool samples using the Fast DNA Spin Kit (MP Biomedicals, USA), following the manufacturer**’**s instructions. The V3 and V4 region of prokariotic 16S rRNA genes were amplified by polymerase chain reaction (PCR), using 16SF (5**’**-CCTACGGGNGGCWGCAG-3**’**) and 16SR (5**’**-GACTACHVGGGTATCTAATCC-3**’**) primers (Klindworth et al., 2013). PCR reactions were carried out in 50 µL, with 25 µL 2X KAPA HiFi HotStart ReadyMix (Roche, Switzerland), 50 ng of DNA and primers to final concentration of 200 nM. Thermal cycling consisted of initial denaturation at 95°C for 3 minutes, followed by 25 cycles of denaturation at 95°C for 30 seconds, annealing at 55°C for 30 seconds, and extension 72°C for 30 seconds. A final extension of 72°C for 5 minutes was applied. The products were analyzed by capillary electrophoresis in Fragment Analyzer™ (Agilent Technologies, USA). Then, libraries preparation was obtained following manufacturer**’**s instructions, quantified in a Quantus Fluorometer (Promega, USA) and analyzed by capillary electrophoresis in Fragment Analyzer™ (Agilent Technologies, USA). Finally, sequencing was performed on a MiSeq sequencer (Illumina, USA), after preparation and loading on a MiSeq Reagent Kit V3 600 cycles following the manufacturer**’**s instructions.

### Community structure analysis

#### Sequence analysis

The quality control of the sequences was carried out using Divisive Amplicon Denoising Algorithm 2 (DADA2) and reads were demultiplexed and processed using Quantitative Insights Into Microbial Ecology 2 (QIIME2) (Caporaso et al., 2010), using amplicon sequence variants (ASVs) for the analysis. For alpha diversity, Faith’s phylogenetic diversity (PD) index was evaluated. PD and the alpha rarefaction curves were obtained through a QIIME2 plugin. For beta diversity, unweighted UniFrac distance was evaluated, applying PERMANOVA with 999 permutations (alpha = 5%) in addition to the Jaccard distance. Principal Coordinates Analysis (PCoA) 3D graphics were generated using EMPeror (Vázquez-Baeza et al., 2013). The Kolmogorov-Smirnov test was applied to determine the statistical significance (alpha = 5%).

### Linear discriminant analysis effect size

LEfSe (Segata et al., 2011) was applied to a table of Features (obtained from QIIME2) to identify potential microorganisms groups that could be associated with hypercholesterolemic individuals and controls. Differentially abundant bacterial groups were defined with a LDA score (log10) over 2. The Kruskal-Wallis test was applied as a factorial test for the classes. The level of significance was alpha = 5%.

### Phylogenetic investigation of communities by reconstruction of unobserved states 2

PICRUSt2 allows for the prediction of the functional abundance of bacterial groups based on 16S rRNA sequencing data. For this analysis, a file of aligned Amplicon Sequence Variants (ASVs) was generated using the QIIME data to obtain gene predictions. Finally, gene family profiles and pathway abundances for samples and groups were obtained (Douglas et al., 2020). Statistical Analysis of Metagenomic Profiles (STAMP) tool was used to visualize the results (Parks et al., 2014).

### Co-occurrence networks analysis

The co-occurrence analysis was based on the methodology proposed by Bradley (Bradley, 2020), which considers the probabilistic co-occurrence model suggested by Veech (2013), establishing the co-occurrence by pairs and delivering the significant interactions (alpha = 5%) (Veech, 2013). For the latter, the coexistence of pairs of species with lower and higher frequencies than what would be expected to be found by chance is taken into account. Finally, the visualization of the results was carried out using visNetwork, based on R, which takes into account the data frames that describe the nodes and edges of the networks.

### Statistical analysis

The data was analyzed using the software GraphPad Prisma v. 8.0 (GraphPad Software Inc., USA). Descriptive statistics were obtained for each variable on the data of all participants. In addition, the biochemical and anthropometric characteristics of the groups were compared using the Mann-Whitney test. The detected phyla of both groups were compared with the Kolmogorov-Smirnov test. Statistical significance was established with a p value less than 0.05.

## Results

### Anthropometric, clinical and biochemical parameters

Anthropometric, clinical and biochemical parameters are summarized in **Table 1**. The mean age between the groups was not significantly different (p=0.525). Similarly, there were no significant differences in SBP, DBP, glucose and HDL-C levels. As expected, triglycerides (p=0.003), total cholesterol (p<0.0001) and LDL-C (p<0.0001) serum levels were significantly higher in hypercholesterolemic individuals. However, weight (p<0.0001) and BMI (p=0.004) were significantly higher in control individuals.

**Table 1:** Anthropometric and biochemical characteristics of hypercholesterolemic individuals and controls.

Parameters	Controls (n=30)	Hypercholesterolemic (n=27)	*P*
Age (years)	60.07 ± 10.27	60.83 ± 5.00	0.525
Height (m)	1.64 ± 0.02	1.58 ± 0.02	0.068
Weight (Kg)	79.34 ± 2.61	64.48 ± 1.83	< 0.0001*
BMI^1^ (Kg/m^2^)	29.72 ± 4.20	25.83 ± 3.21	0.004*
SBP^2^ (mmHg)	128.00 ± 16.77	123.00 ± 10.19	0.765
DBP^3^ (mmHg)	81.29 ± 13.97	80.42 ± 8.93	0.970
Glucose (mmol/L)	5.88 ± 0.79	5.79 ± 0.72	0.688
Triglycerides (mmol/L)	1.73 ± 0.97	2.26 ± 0.93	0.003*
Total Cholesterol (mmol/L)	4.40 ± 0.53	7.55 ± 0.73	< 0.0001*
HDL^4^-Cholesterol (mmol/L)	1.51 ± 0.31	1.58 ± 0.40	0.827
LDL^5^-Cholesterol (mmol/L)	2.24 ± 0.41	5.23 ± 0.74	< 0.0001*

^1^ BMI, Body mass index; ^2^ SBP, Systolic blood pressure; ^3^ DBP, Diastolic blood pressure; ^4^ HDL, High-density lipoprotein; ^5^ LDL, Low-density lipoprotein. Mann-Whitney test. * P-value <0.05.

### Community structure analysis

#### Sequence analysis

**Figure 1:**
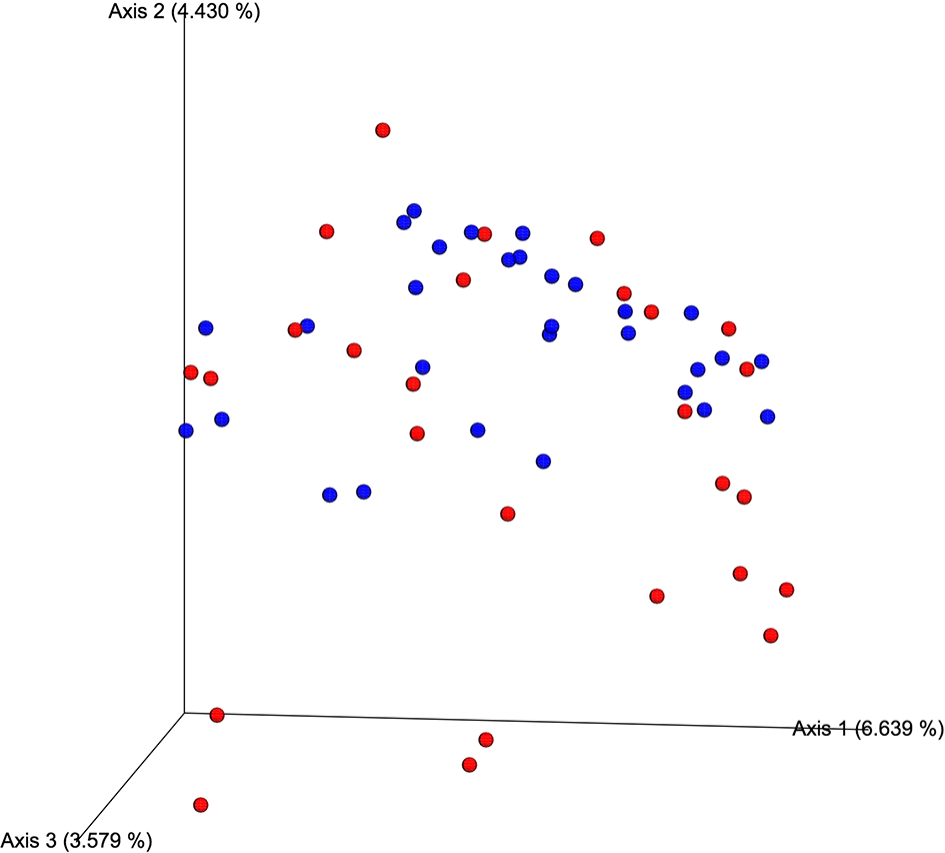
Qualitative measurement of the dissimilarity (Jaccard distance) of the microbial communities associated with of hypercholesterolemic individuals and controls. In the Principal Coordinates Analysis (PCoA), produced by EMPeror, hypercholesterolemic individuals are indicated in red, while those control individuals are indicated in blue.

The average number of sequences obtained in the MiSeq platform was 318,242 reads per individual. The total number of reads obtained was 18,776,291. There were no significant differences between hypercholesterolemic individuals and controls respect to the evaluation of alpha (Faith’s PD index, p=0.396) and beta (unweighted UniFrac distance, p=0.133) diversity. Jaccard distance, indicated in the **Figure 1**, shows that for most of the samples analyzed there is no dissimilarity in the microbial communities, with the exception of some cases of hypercholesterolemic participants (red dots), at the bottom of the graph.

**Figure 2:**
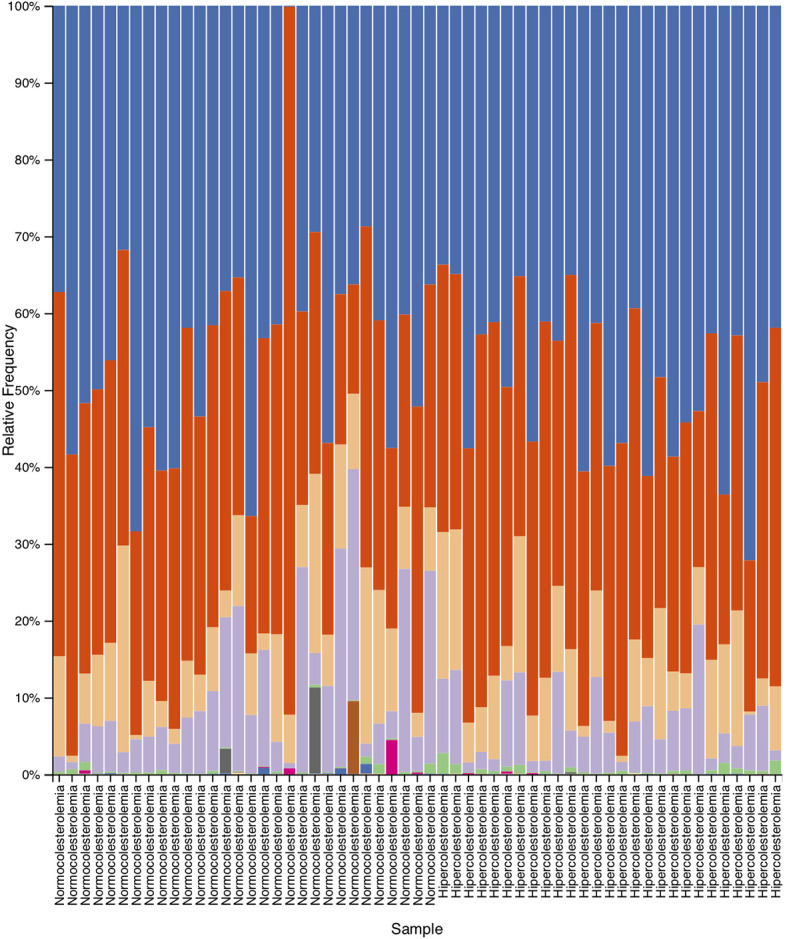
Relative abundance obtained by taxonomic analysis with Quantitative Insights Into Microbial Ecology 2 (QIIME2), which indicate the different phylum present in the deposition samples of hypercholesterolemic individuals and controls.

As results of the taxonomic analysis, **Figure 2** represents the relative abundance of the different microbial communities of hypercholesterolemic individuals and controls. No significant differences were observed in most of the phylum, excepting Actinobacteria (p=0.025), predominantly present in hypercholesterolemic individuals. The relative abundance of Bacteroidetes was 50.63% in hypercholesterolemic individuals and 47.72% in controls (p=0.845). Firmicutes accounted for 35.57% and 43.66% of the microbial taxa in hypercholesterolemic individuals and controls (p=0.279), respectively. The complete microbial profile is presented in **Table 2**.

**Table 2:** Bacterial phylum detected in fecal samples of hypercholesterolemic individuals and controls.

	Controls (n=30)	Hypercholesterolemic (n=27)	*P*
% Bacteroidetes	47.72 ± 15.44	50.63 ± 13.97	0.845
% Firmicutes	43.66 ± 15.10	35.57 ± 10.99	0.279
% Bacteria	2.15 ± 3.51	5.83 ± 6.21	0.105
% Proteobacteria	4.99 ± 6.24	5.58 ± 4.53	0.402
% Actinobacteria	0.09 ± 0.24	0.36 ± 0.60	0.025*
% Elusimicrobia	0.66 ± 3.04	0	–
% Fusobacteria	0.11 ± 0.61	0	–
% Cyanobacteria	1.01 ± 0.21	0.19 ± 0,12	0.245
% Lentisphaerae	0.30 ± 1.12	0	–
% Synergistetes	–	–	–
% Euryarcheota	–	–	–
% Verrucomicrobia	0.16 ± 0.50	0.01 ± 0.07	0.199

Kolmogorov-Smirnov test. * P-value <0.05.

### Differentially abundant bacterial groups present in hypercholesterolemic and controls

**Figure 3:**
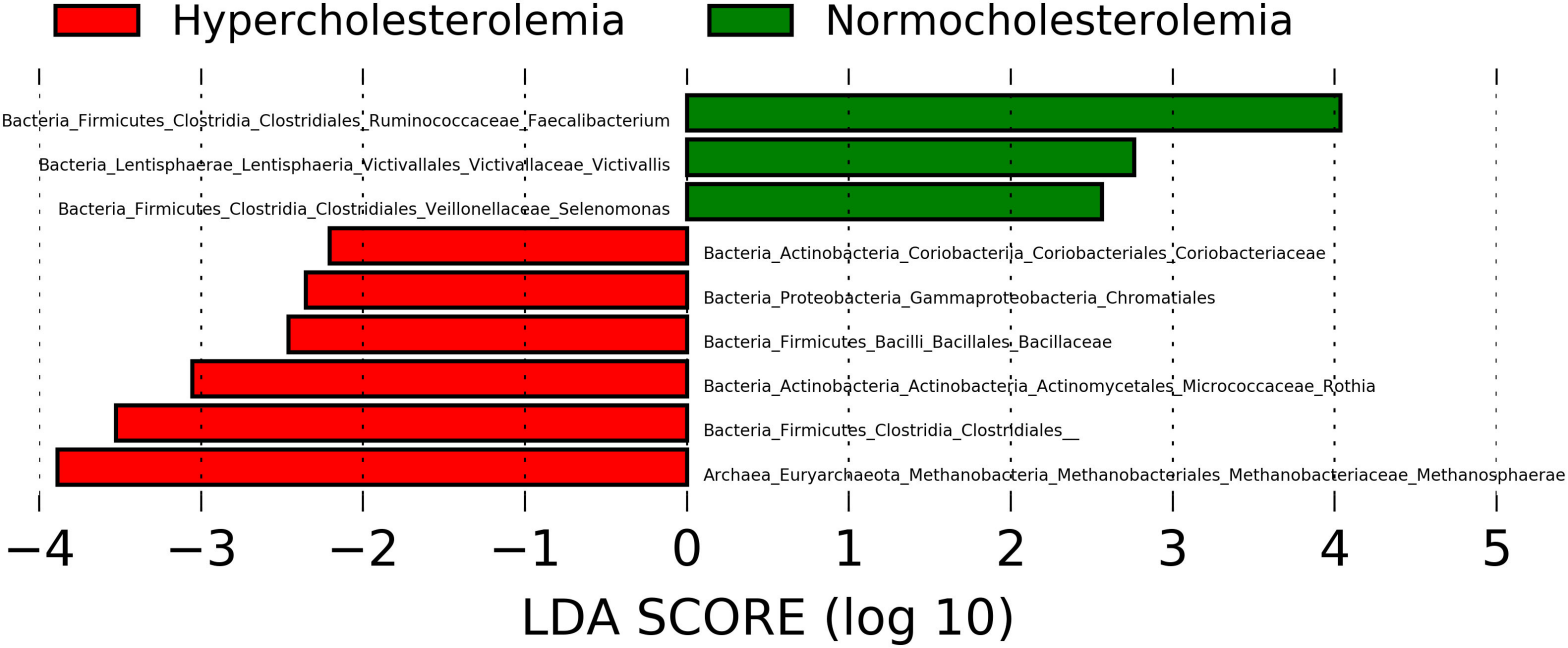
Microbial taxa with Linear discriminant analysis (LDA) score greater than 2 present in hypercholesterolemic individuals (red) and controls (green). P-value <0.05.

The LEfSe comparison between the stool microbiota of hypercholesterolemic individuals and controls suggests the presence of some specific taxa to each group. The genera *Faecalibacterium*, *Victivallis* and *Selenomonas* were likely associated with controls subjects, while the microbiota of participants with hypercholesterolemia contains unique archaeal and bacterial taxa, such as *Methanosphaera* and *Rothia*, along with representative taxa from Coriobacteriaceae and Bacillaceae families and Chromatiales and Clostridiales orders (**Figure 3**).

### Functional abundance prediction of bacterial groups in hypercholesterolemic and controls

**Figure 4:**
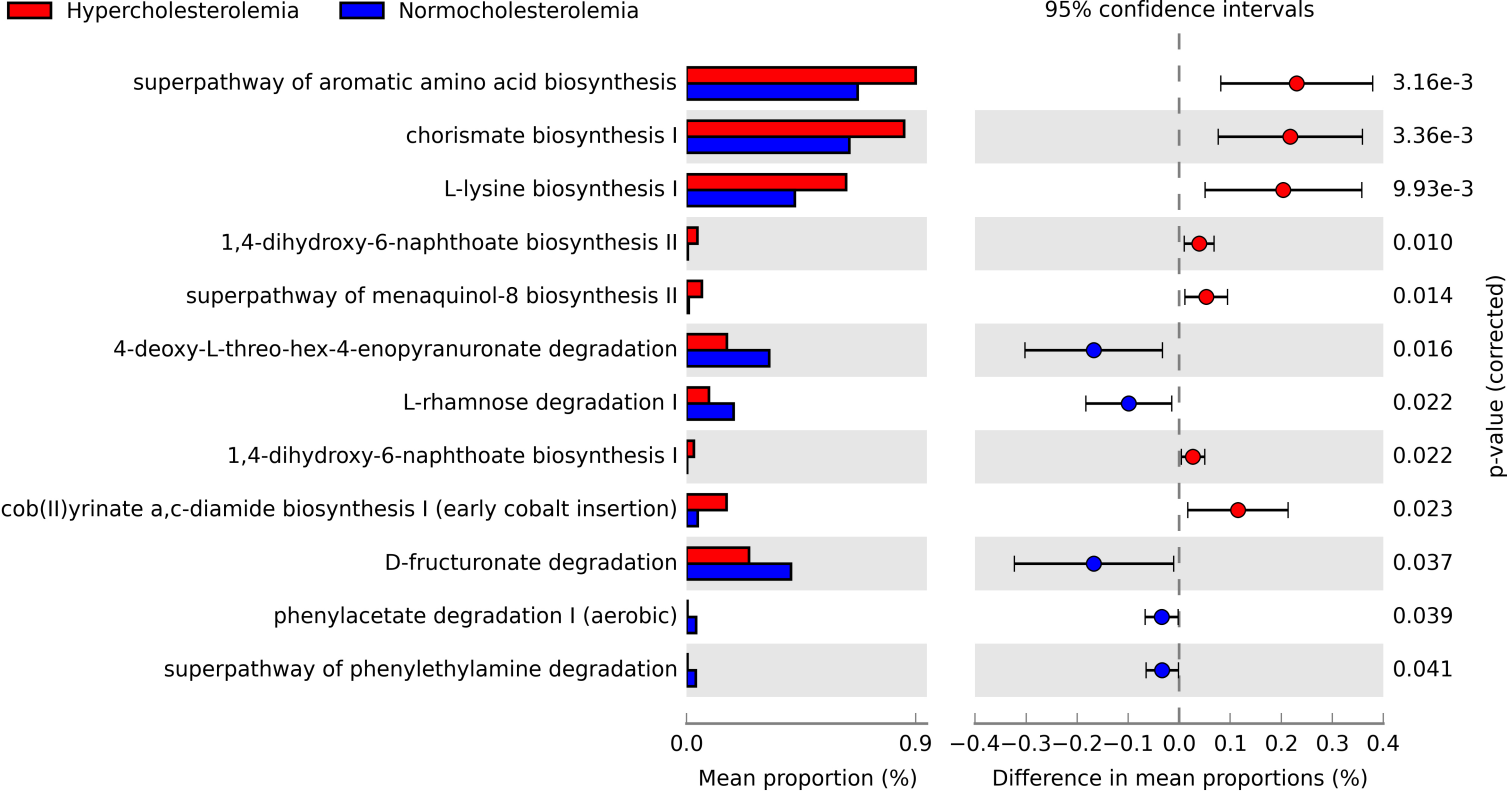
Pathways obtained through PICRUSt2, which are significantly associated with hypercholesterolemic individuals and controls. The presence of biosynthetic pathways predominates in hypercholesterolemic individuals, while degradation pathways are more relevant in controls. P-value <0.05.

Hypercholesterolemic individuals showed the predominant presence of biosynthesis pathways, including the super-pathway of aromatic amino acid biosynthesis (p=0.00316), chorismate biosynthesis I (p=0.00336), L-lysine biosynthesis I (p=0.00993), 1-4-dihydroxi-6-naphthoate biosynthesis I (p=0.022) and II (p = 0.010), menaquinol-8 biosynthesis II super-pathway (p = 0.014) and cob(II)yrinate a,c-diamide biosynthesis I (early cobalt insertion) (p=0.023). The first three are the pathways with the highest proportion in this study group (close to 0.9%). While in controls, degradation pathways are highlighted, such as 4-deoxy-L-threo-hex-4-enopyranuronate degradation of L-rhamnose degradation I (p=0.022), D-fructuronate degradation (p=0.037), phenylacetate degradation I (aerobic) (p=0.039), and phenylethylamine degradation pathway (p=0.041) (**Figure 4**).

### Interactions of the different bacterial groups present in hypercholesterolemic and controls

**Figure 5:**
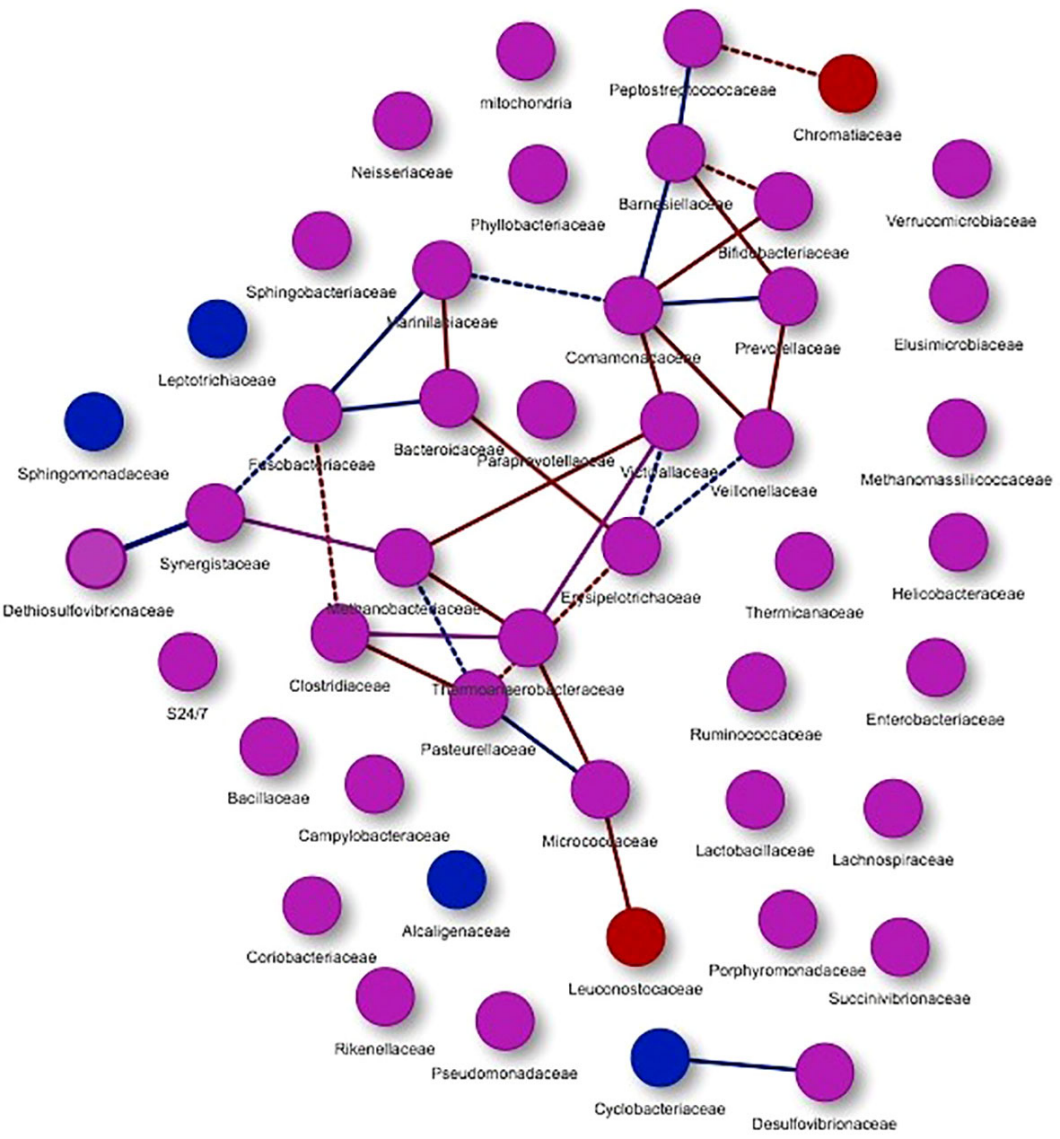
Co-occurrence networks of microorganisms present in the study groups. Purple nodes represent the presence of microorganisms in both groups, while red nodes are mainly associated with hypercholesterolemic individuals and blue nodes with controls. With the same colors the interactions or edges are evidenced, associating these to the study groups. Continuous lines (edges) refer to co-occurrences that occur between microorganisms with a higher frequency than expected, while dashed lines correspond to interactions with less frequency than expected. P-value <0.05.

Co-occurrence networks allow to explore the interactions between different groups of microorganisms. This analysis was carried out at a general level (considering the two studied groups) as well as each study group separately. **Figure 5** shows the graphic representation of the co-occurrence considering all the microorganisms of both studied groups.

Through this analysis, 47 nodes were obtained (**Figure 5**), presenting co-occurrence interactions mainly associated with microorganisms from the groups Peptostreptococcaceae, Chromatiaceae, Barnesiellaceae, Bifidobacteriaceae, Comamonadaceae, Prevotellaceae, Marinilabiaceae, Fusobacteriaceae, Bacteroidaceae, Victivallaceae, Veillonellaceae, Dethiosulfovibrionaceae, Synergistaceae, Methanobacteriaceae, Erysipelotrichaceae, Clostridiaceae, Thermoanaerobacteraceae, Pasteurellaceae, Micrococcaceae and Leuconostocaceae. For these nodes, co-occurrence relations with a greater tendency in hypercholesterolemic individuals (red edges) and in other cases for controls (blue edges) can be observed. In addition, other microbial groups are presented, but these do not show evident interaction in this analysis.

**Figure 6:**
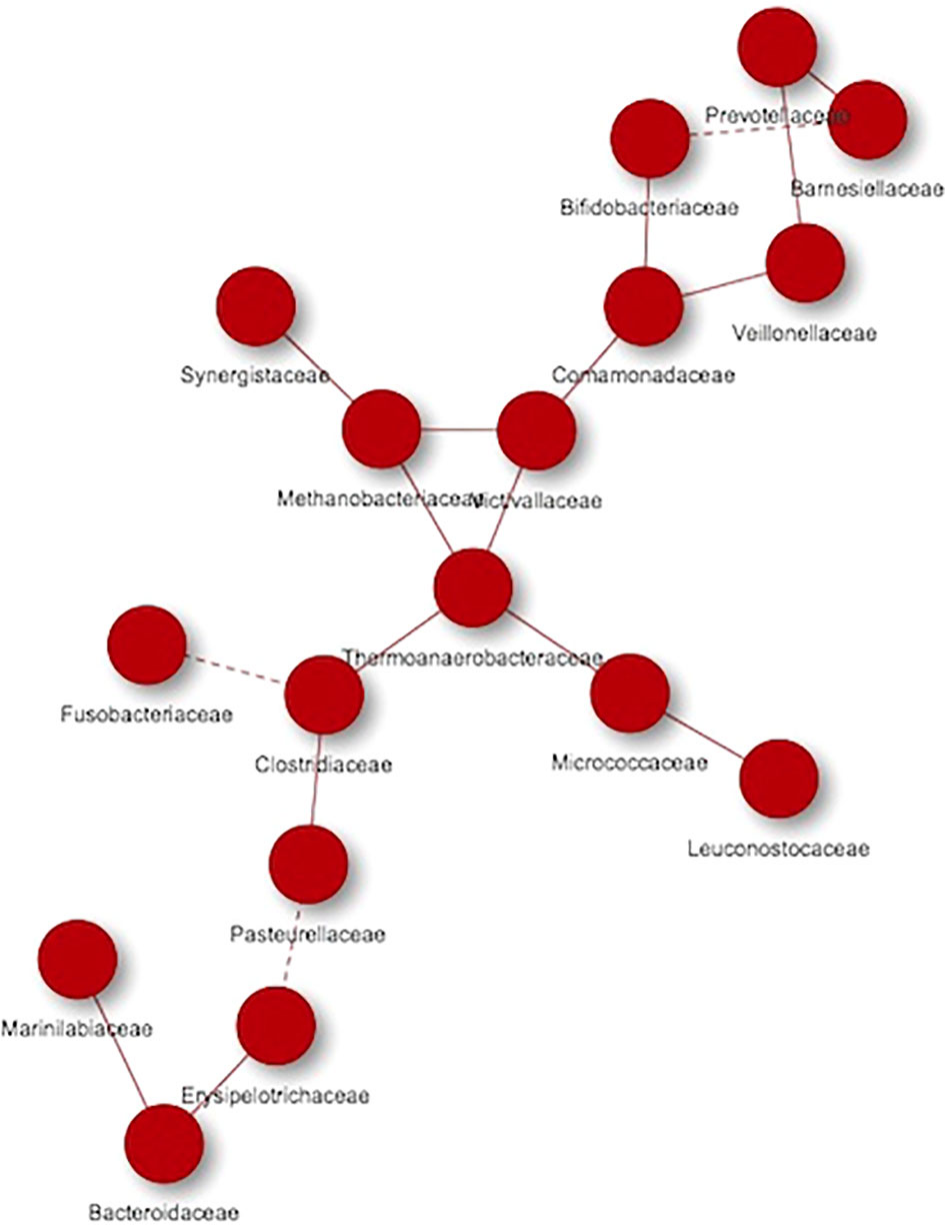
Co-occurrence networks of microorganisms present in hypercholesterolemic individuals. Continuous lines (edges) refer to co-occurrences that occur between microorganisms with a higher frequency than expected, while dashed lines correspond to interactions with less frequency than expected. P-value <0.05.

When performed the analysis separately, **Figure 6** showed the microorganisms that were mainly associated with the hypercholesterolemic group, thus obtaining 17 nodes, grouped into a single network, where high and low frequency interactions are present (continuous and dashed lines, respectively). In this study group, we found Prevotellaceae, Bifidobacteriaceae, Barnesiellaceae, Veillonellaceae, Comamonadaceae, Victivallaceae, Methanobacteriaceae, Synergistaceae, Thermoanaerobacteraceae, Micrococcaceae, Leuconostocaceae, Clostridiaceae, Fusobacteriaceae, Pasteurellaceae, Erysipelotrichaceae, Bacteroidaceae and Marinilabiaceae. In some cases, a microorganism can present both types of interactions depending on the group of microorganisms with which it is related, such as the Clostridiaceae group, which has a high frequency co-occurrence when it interacts with Pasteurellaceae or Thermoanaerobacteraceae, while the interaction is low when it is interacting with Fusobacteriaceae.

**Figure 7:**
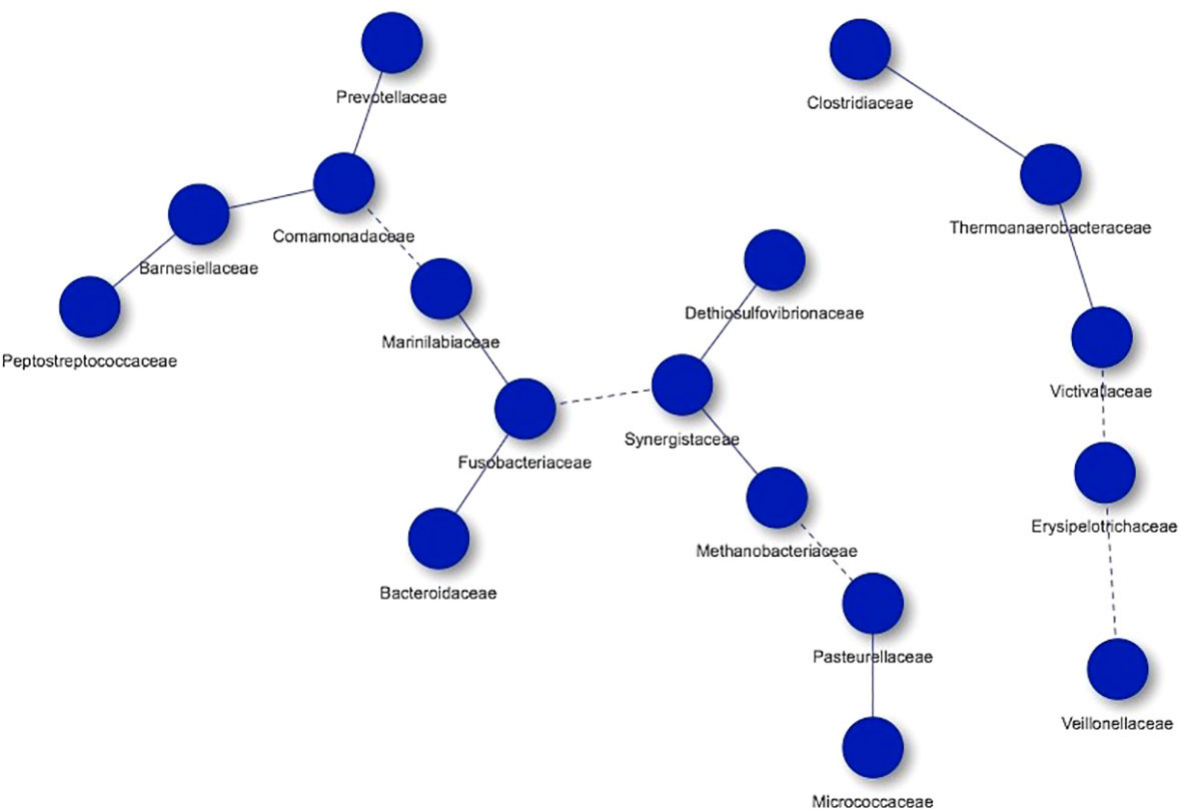
Co-occurrence networks of microorganisms present in controls. Continuous lines (edges) refer to co-occurrences that occur between microorganisms with a higher frequency than expected, while dashed lines correspond to interactions with less frequency than expected. P-value <0.05.

Regarding the controls, 17 nodes were also obtained, organizing them in two networks, with the most of microorganisms participating in one of them. In this study group we found Clostridiaceae, Thermoanaerobacteraceae, Victivallaceae, Erysipelotrichaceae, Veillonellaceae, Prevotellaceae, Comamonadaceae, Barnesiellaceae, Peptostreptococcaceae, Marinilabiaceae, Fusobacteriaceae, Bacteroidaceae, Synergistaceae, Dethiosulfovibrionaceae, Methanobacteriaceae, Pasteurellaceae and Micrococcaceae. As in the group of hypercholesterolemic individuals, there are co-occurrences with high and low frequencies of interaction (**Figure 7**).

## Discussion

Considering that hypercholesterolemia is a main modifiable risk factor of CVDs, it is relevant to analyze a potential association of the different microbial taxa present in the intestinal tract with individuals classified as hypercholesterolemic and normocholesterolemic by LDL-C levels. Here, we profiled the stool microbiota of 57 adult subjects in order to identify possible associations between the composition of the microbiota and the LDL-C levels of these individuals.

By analyzing the sequences of the 16S rRNA gene, it was possible to establish that there were no significant differences at alpha and beta diversity between the studied groups (Faith’s PD index, p=0.396 and the unweighted UniFrac distance, p=0.133, together with the Jaccard distance). This contrasts with other works, where the difference in diversity between groups generally stands out, which can also be accompanied by differences in the abundance of microbial groups (Turnbaugh et al., 2009; Hansen et al., 2019).

Through the taxonomic analysis, highlights the presence of phylum Actinobacteria (p=0.025) in hypercholesterolemic individuals. This bacterial group being already indicated in other studies as part of the intestinal microbiota in humans, together with Firmicutes, Bacteroidetes and Proteobacteria phylum (Turnbaugh et al., 2009; Binda et al., 2018). Bacteroidetes and Firmicutes stand out predominantly, and our observations are consistent with this. The Bacteroidetes/Firmicutes relation has been related to the predisposition to certain diseases, with a lower abundance of Bacteroidetes being observed in obese individuals, in which bacteria belonging to the Firmicutes group abound (Ley et al., 2006; Gomes et al., 2018). Although in our study the BMI was increased in controls compared to hypercholesterolemic individuals (p=0.004), no differences were found between Bacteroidetes and Firmicutes (p=0.845 and p=0.279, respectively) by the taxonomic analysis. However, this remains controversial, since The Human Microbiome Project Consortium (2012) found a poor association between the Bacteroidetes/Firmicutes ratio and BMI. Furthermore, a higher abundance of Bacteroidetes in the gut microbiota has been associated with more industrialized populations compared to developing populations (Yatsunenko et al., 2012).

As expected, significant differences in the LDL-C (p<0.0001), triglycerides (p=0.003) and total cholesterol (p<0.0001) levels were observed in hypercholesterolemic compared to controls. Fu et al. (2015), suggest that the gut microbiome plays an important role in blood lipid levels, highlighting significant variations in triglyceride levels (2.74% variation) and HDL-C (3.83% variation). In contrast to our results, these authors showed a little impact of microbial groups on LDL-C (0.01% variation) and total cholesterol (0.01% variation) levels. Even so, the evidence of different studies reveals a close relation of the intestinal microbiota with lipids and lipoproteins, as well as with liver function and some of its alterations (Yu et al., 2019).

The intestinal microbiota obtains its nutrients from the host diet. Thus, *Bacteroides, Roseburia, Bifidobacterium, Faecalibacterium*, and *Enterobacteria* have been shown to be capable of fermenting undigested carbohydrates and producing short-chain fatty acids (SCFA), such as acetate, propionate, and butyrate (Al-Lahham et al., 2010). Both butyrate and propionate have low systemic concentrations whereas acetate levels are higher (Schoeler and Caesar, 2019). After absorption, SCFAs can induce lipogenesis and increase triglyceride stores through molecular pathways (Tokarek et al., 2021). Related to this, Granado-Serrano et al. (2019) founded high levels of SCFAs such as isobutyric and isovaleric acids in hypercholesterolemic individuals. In addition, the intestinal microbiota may interact with lipid metabolism through inhibition of hepatic cholesterol synthesis, redistribution of plasma cholesterol to the liver by the action of SCFAs, and/or deconjugation of bile acids by hydrolysis (Sonnenburg et al., 2006). In conjunction with evidence showing that oral supplementation with probiotics can reduce LDL-cholesterol concentration, lowering atherogenic indices and improving glycemic control (Ejtahed et al., 2012). However, we did not have access to data related to the diet of the participants, which we consider a limitation of this study that will be solved in future analyzes, in which the study of SCFAs might be included, considering that several microbial groups present in the studied individuals are related to the profile of these molecules.

In our study, LEfSe shows an enrichment of *Faecalibacterium* associated with controls individuals. *Faecalibacterium prausnitzii* is regarded as a butyrate producer in the gut, and it is used to treat dysbiosis in diseases that have an inflammatory component (Ganesan et al., 2018). This bacterial genus is recognized as a marker of gut health (Laursen et al., 2017) and changes in its abundance are associated with ulcerative colitis, colorectal cancer, and other diseases (Benevides et al., 2017). Other bacterial genera enriched in control individuals are *Victivallis* and *Selenomonas*. The genus *Victivallis* is recognized for being naturally present in gut (van Passel et al., 2011), though its abundance has been reported to vary with diet. Zhang et al. (2019), for instance, observed an increasing trend of abundance of this taxon in individuals treated with resistant starch, a prebiotic dietary fiber, also observing a decrease in LDL-C in the studied individuals. This would be concordant with the LDL-C levels and the presence of the genus *Victivallis* in the studied individuals. Regarding the genus *Selenomonas*, this has been mainly related to the oral health of children (Mashima et al., 2017), likewise it has been related to long-standing diseases that have an important inflammatory component, such as colorectal cancer and systemic lupus erythematosus (SLE) (Correa et al., 2017; Allali et al., 2018). In addition, some species such as *Selenomonas noxia* have been related to overweight (Goodson et al., 2009), also observed in the control group.

In hypercholesterolemic subjects, several ASVs were also differently abundant. Chromatiales and Clostridiales orders present genera and species that positively and negatively impact the development of diseases linked to the gut microbiota. Although, Chromatiales can be recognized as belonging to the Proteobacteria phylum, that are related to diverse pathologies including gut inflammation, irritable bowel syndrome and metabolic syndrome (Shin et al., 2015; Litvak et al., 2017). Regarding Clostridiales, it is a diverse group in which the genus *Clostridium* and some of its species are the most outstanding. *Clostridium difficile*, is known as a pathogenic agent associated with gut microbiota dysbiosis (Antharam et al., 2016), but other species such as *Clostridium coccoides* may also occur. Jamar et al. (2018), demonstrated the considerable presence of *C. coccoides* in individuals with metabolic syndrome, highlighting the effect of microbiota alterations on high levels of triglycerides in obese patients. Considering that hypercholesterolemic subjects in our study also show high levels of triglycerides, we hypothesize that would be related to the enrichment of these taxa in their gut microbiota.

The archaea of the genus *Methanosphaera* was also significantly associated to hypercholesterolemia in our study. These microorganisms have the capacity to produce methane in the presence of H_2_ and methanol (Gaci et al., 2014; Lurie-Weinberger and Gophna, 2015). It has been suggested that colonization by archaea is potentially detrimental to host health, due to alterations in intestinal metabolism and synthropic interactions with other microorganisms, which would stimulate the production of certain SCFAs (van de Pol et al., 2017; Canfora et al., 2019). Other studies demonstrate the importance of archaea such as *Methanosphaera stadtmanae* in the reduction of trimethylamine (TMA) at the gut level, which likely leads to a reduction in trimethylamine-N-oxide (TMAO) concentration (Gaci et al., 2014). In contrast to the groups of microorganisms indicated above, in relation to the genus *Rothia*, and the families Bacillaceae and Coriobacteriaceae, no data were found to establish a relationship between them and cholesterol metabolism.

The PICRUSt2 analysis showed multiple pathways differentially present in hypercholesterolemic individuals compared to controls. The most significant were the superpathway of aromatic aminoacid biosynthesis (p=0.00316), chorismate biosynthesis I (p=0.00336) and L-lysine biosynthesis I (p=0.00993). These three pathways are associated with the metabolism of amino acids. The production of amino acids and other peptides is part of the molecules generated by bacteria of the gut microbiota on a regular basis, so no evidence has been found that increased production of these molecules is related to hypercholesterolemia or associated pathologies. However, it has been possible to associate the levels of indoxyl sulfate levels with coronary atherosclerosis, as it has also been possible to associate p-cresyl sulfate with the prediction of some cardiovascular event, both metabolic derivatives of aromatic amino acids tryptophan and tyrosine, respectively, both produced by the intestinal microbiota (Wang and Zhao, 2018). Regarding the microorganisms related to the metabolism of amino acids, various groups such as Firmicutes, Bacteroidetes, among others, present a strong association with these biomolecules, such as the Clostridiaceae family, which presents a positive association with the super pathways associated with amino acids, being one of the groups of microorganisms identified in our study (Murga-Garrido et al., 2021).

Regarding pathways significantly associated with controls, we observed differences in 4-deoxy-L-threo-hex-4-enopyranuronate degradation (p=0.016), L-rhamnose degradation I (p=0.022) and D-fructuronate degradation (p=0.037). Unlike the group of hypercholesterolemic individuals, these pathways are mainly related to carbohydrate metabolism. Considering that gut microorganisms have the ability to digest simple and complex carbohydrates (Coker et al., 2021), it is likely that the significant presence of these pathways in this group would be related to a balanced diet in which carbohydrates associated with fiber predominate. However, no data about dietary intake was recorded in the studied individuals.

The application of co-occurrence analysis to the different microorganisms identified in the studied groups allows us to establish possible interactions (edges) among them, although these analyzes correspond to a bioinformatic approximation and require confirmation by other methodologies. In both hypercholesterolemic and control individuals, most co-occurring interactions occur more frequently than expected (continuous lines), compared to those that occur less frequently than expected (dashed lines). An example to highlight in terms of interactions with greater frequency than expected is the case of the Clostridiaceae family (Firmicutes) with other microbial groups (in individuals with hypercholesterolemia). Particularly, in this case, the negative association and absence of direct interaction between this family and the Bacteroidaceae (Bacteroidetes) family should be highlighted, which reflects a similar result described above for these two taxa (Faust et al., 2012). In general, the proper presence and interaction of the microbiome is crucial to maintain the health in hosts, from the first years of life to old age, allowing the development of adequate biological and immunological processes (Thomas et al., 2017).

Given the level of evidence that exists about the microorganisms identified and exposed in this article, and considering their possible association with cholesterol metabolism, would be interesting to investigate them more in depth to understand their impact on the production and regulation of cholesterol in the organism, especially considering that studies in mice have shown that the management of the microbiota together with classical cholesterol therapies can contribute to the prevention of diseases such as non-alcoholic fatty liver disease (NAFLD)-associated hepatocellular carcinoma (X. Zhang et al., 2021), as well as the association between gut microbiota and the effect of the lipid-lowering therapy using statins (Zimmermann et al., 2020); it is of great importance to obtain new evidence that contributes to the diagnosis and treatment of hypercholesterolemia, since its correspond to a one of main modifiable risk factors for cardiovascular diseases.

## Data availability statement

The data presented in the study are deposited in the BioProject repository, accesion number PRJNA842179.

## Ethics statement

The studies involving human participants were reviewed and approved by Comité Ético Científico, Universidad de La Frontera. The patients/participants provided their written informed consent to participate in this study.

## Author contributions

NS and CM conceptualized and wrote the final manuscript. CM performed the experiments and obtained the results of the procedures. MR-H, RA-C, and SC-O analyzed the results using software. NS, GR, and CR. validated the results obtained. VM-C, KS, and PL carried out the formal analysis of the manuscript. NS, FL, and LS managed the resources used. NS, VM-C and PL obtained the financial support for the project. All authors contributed to the article and approved the submitted version.

## Funding

This research was supported by FONDECYT-ANID (11160364 to NS; 1211731 to VMC); FONDAP-ANID (15130011 to VMC); and by the ANID PhD fellowship (21181271 to CM).

## Conflict of interest

The authors declare that the research was conducted in the absence of any commercial or financial relationships that could be construed as a potential conflict of interest.

## Publisher’s note

All claims expressed in this article are solely those of the authors and do not necessarily represent those of their affiliated organizations, or those of the publisher, the editors and the reviewers. Any product that may be evaluated in this article, or claim that may be made by its manufacturer, is not guaranteed or endorsed by the publisher.

## Abbreviations

CVD, Cardiovascular disease; CAD, Coronary artery disease; LDL-C, Low-density lipoprotein cholesterol; PCR-DGGE, Polymerase chain reaction-gel electrophoresis with denaturing gradient; SBP, Systolic blood pressure; DBP, Diastolic blood pressure; HDL-C, High-density lipoprotein cholesterol; BMI, Body mass index; LDA, Linear Discriminant Analysis; PCoA, Principal Coordinates Analysis; PD, Phylogenetic diversity; SLE, Systemic lupus erythematosus; ASV, Amplicon Sequence Variant; SCFAs, Short-chain fatty acids; TMA, Trimethylamine; TMAO, Trimethylamine-N-oxide; NAFLD, Non-alcoholic fatty liver disease; NCEP, National Cholesterol Education Program; PCR, Polymerase chain reaction; DADA2, Divisive Amplicon Denoising Algorithm 2; LEfSe, Linear Discriminant Analysis Effect Size; QIIME2, Quantitative Insights Into Microbial Ecology 2; PICRUSt2, Phylogenetic Investigation of Communities by Reconstruction of Unobserved States 2; STAMP, Statistical Analysis of Metagenomic Profiles.
